# The Clinical Course of Paranasal Sinus Mucocoeles—A Retrospective Case Series

**DOI:** 10.3390/jcm13020397

**Published:** 2024-01-11

**Authors:** Shyam Gokani, Kala Kumaresan, Sushritha Adari, Mehmet Ergisi, Oladapo Oludeye, Lavandan Jegatheeswaran, Carl Philpott

**Affiliations:** 1 Rhinology and ENT Research Group, Norwich Medical School, University of East Anglia, Norwich NR4 7TJ, UK; drkalakumaresan@gmail.com (K.K.); m.ergisi@uea.ac.uk (M.E.); c.philpott@uea.ac.uk (C.P.); 2 Department of Ear, Nose and Throat Surgery, James Paget University Hospital, Great Yarmouth NR31 6LA, UK; sushritha7494@gmail.com (S.A.); oladopo.ouldeye@jpaget.nhs.uk (O.O.); lavandan.jegatheeswaran@jpaget.nhs.uk (L.J.)

**Keywords:** chronic rhinosinusitis, rhinology, rhinosinusitis complications, endoscopic sinus surgery, paranasal sinus mucocoele

## Abstract

Paranasal sinus mucocoeles can be secondary to chronic rhinosinusitis and can result in intra-orbital and intra-cranial complications requiring surgical management. The natural history of conservatively managed mucocoeles is not well established. We aimed to quantify the proportion of radiologically identified paranasal sinus mucocoeles resulting in complications over 10 years. We retrospectively reviewed anonymised data on radiologically diagnosed mucocoeles between 2011 and 2021 at two UK hospitals. We collected data on age at presentation, extent of sinus involvement, management and complications. We identified 60 patients with mucocoeles, of which 35 (58%) were incidental findings from radiological investigations. The mean age was 58 years. Fifteen patients (25%) were managed surgically and one presented with recurrence following surgery. Overall, six patients (10%) had an intra-orbital extension of their mucocoele and three (5%) had an intra-cranial extension. There was no difference in the rates of intra-cranial extension between conservative and surgical cases but surgical cases included a higher rate of intra-orbital extensions (27% vs. 4%, *p* = 0.01). The proportion of patients requiring surgical intervention in this study is low. Incidental and asymptomatic mucocoeles have a relatively benign disease course and selected uncomplicated cases can be considered for conservative management with serial scanning at 12 months.

## 1. Introduction

Paranasal sinus mucocoeles are non-malignant, expansive and mucus-filled cysts lined with pseudostratified columnar epithelium. A vast proportion arise in the frontal and frontoethmoidal regions [1]. Their incidence is equal in males and females and most prevalent in those aged between 40 and 60 years old [2]. Mucocoeles form due to sinus outflow tract obstruction either idiopathically, most commonly, or secondary to chronic rhinosinusitis, previous endoscopic sinus surgery, chronic inflammatory processes, previous trauma, or disorders of mucociliary clearance [3,4,5]. Following outflow tract obstruction, mucous production continues and the cyst-like structure steadily enlarges. This results in a typically insidious onset of symptoms, which can be sub-categorised into neurological, rhinological and ophthalmological in nature. These symptoms include headache, nasal obstruction and abnormal ocular mobility respectively. Symptoms relate to the anatomical location of the affected sinuses. For example, involvement of the frontal and ethmoidal sinuses may result in the presentation of proptosis and reduced ocular mobility. The anatomical proximity of the orbit, coupled with the thin wall of the lamina papyracea, likely explains the relatively common incidence of ophthalmic manifestations in patients with paranasal sinus mucocoeles. These include periorbital pain and swelling, diplopia, decreased ocular motility and globe displacement [6].

Mucocoele enlargement may encroach upon and erode adjacent bone through direct pressure [1]. Furthermore, expansion of the mucocoele in conjunction with an infection can stimulate the host immune system, in particular monocytes and lymphocytes, to trigger a chronic inflammatory response, resulting in the stimulation of cytokine, prostaglandin and collagenase release [7]. The inflammatory response ultimately augments the risk of severe complications from an intra-orbital or intra-cranial extension, namely, meningitis, intra-cranial abscesses, fistulas and compression of local structures.

The differential diagnosis of paranasal sinus mucocoeles includes a number of inflammatory, neoplastic and congenital cystic conditions [8]. The diagnosis necessitates a thorough history exploring the aforementioned risk factors. Following physical examination, radiological findings are crucial for diagnosis. Computed tomography (CT) is used as the primary choice of imaging since it can assess the extent of mucocoele expansion as well as the structure and involvement of local anatomy. CT provides great benefit in that it displays the degree of bone erosion and, therefore, a possible intra-orbital or intra-cranial extension. Common findings include sinus opacification, with thinning or erosion of the sinus wall [3]. Magnetic resonance imaging provides an alternative to CT, with particular utility in delineating paranasal sinus mucocoeles from paranasal sinus carcinomas and soft tissue [8].

Definitive management is surgical, traditionally through external approaches such as the Lynch–Howarth incision, frontal sinus osteoplastic flaps, or the Caldwell-Luc procedure. Endoscopic marsupialisation is now favoured at our centre where possible, providing reduced hospital stay and greater preservation of bone, thereby resulting in lower rates of relapse, morbidity and mortality [9]. The endoscopic approach begins with the patient in the supine position and the head elevated up to 45 degrees, with both nasal cavities examined [10]. In the cavity with pathology, the middle turbinate is displaced to allow visualisation of the mucocoele, and punch forceps are used to open and drain the mucocoele sac. After samples of the sac content are taken for cytology, microscopy, culture and sensitivity, a drain is inserted to allow marsupialisation, which works by preventing premature closure and recurrence of the mucocoele, supported by the placement of antibiotic and steroid gauze preparations into the site. There are cases for which endoscopic surgery alone is ill advised, such as laterally placed frontal mucocoeles and those developing secondary to malignancy, for which external approaches are still recommended [8,11,12]. Irrespective of approach, the goal of surgical intervention is to restore the natural drainage pathway of the sinus involved and improveme a patient’s health-related quality of life. Image-guided navigation systems are also now used at our centre to provide targeted endoscopic surgery.

### 1.1. Rationale

Surgical treatment is clearly indicated in the case of symptomatic mucocoeles or those with complications. However, the role of surgery in asymptomatic and incidental disease is less certain due to limited data on the natural history of mucocoeles. Previous case reviews describe surgical management for the majority of mucocoeles, including asymptomatic patients [1,9,13,14]. Since any sinus surgery involves risks, this approach may result in overtreating some patients. Studies of the conservative management of paranasal sinus mucocoeles are limited but can inform the potential avoidance of surgery, facilitating appropriate resource use and better informing patients of the pros and cons of conservative vs. surgical management. Data on the natural progression of conservative and surgically managed mucocoeles would further our understanding of this topic.

### 1.2. Objective

To document the natural history of paranasal sinus mucocoeles managed conservatively compared to those managed surgically, over a 10-year period at two UK hospitals.

## 2. Materials and Methods

### 2.1. Study Design and Setting

In this consecutive clinical case series, we retrospectively reviewed electronic clinical records for all patients with radiologically identified paranasal sinus mucocoeles across two hospitals: James Paget University Hospital and Norfolk and Norwich University Hospital. Both hospitals provided secondary or tertiary-level rhinology care. Records were reviewed from 1 January 2011 to 31 December 2021 at James Paget University Hospital and 1 April 2014 to 31 December 2021 at Norfolk and Norwich University Hospital. The discrepancy in dates was due to an electronic data system migration preventing access to prior records at Norfolk and Norwich University Hospital.

### 2.2. Participants

Cases were identified from radiology reports of contrast and non-contrast enhanced CT sinus, head and facial bone scans on the national picture archiving and communication system (PACS).

### 2.3. Inclusion Criteria

All reports containing the terms ‘mucocoele’ or ‘mucocele’ were reviewed for inclusion if the mucocoele was present at the time of the scan in the frontal, ethmoid, maxillary or sphenoid sinuses.

### 2.4. Exclusion Criteria

Records were excluded if they described:

- Nasolacrimal sac mucocoele alone;
- Resolved paranasal sinus mucocoeles managed at an alternative centre;
- Chronic rhinosinusitis (CRS) without mucocoele;
- Radiologically suspected mucocoele that was subsequently excluded clinically.

### 2.5. Main Outcome Measures, Variables and Data Sources

Anonymised data were collected from patients’ electronic clinic letters and imaging for age (in years), gender, site of sinus involved, management, complications and indication for imaging. Records were reviewed from the time of diagnosis until 31 December 2021.

### 2.6. Statistical Analysis

The sample mean and standard deviation of age in years were calculated for all patients. The percentage of patients presenting with individual sinus pathology, intra-orbital or intra-cranial extension, and recurrence was calculated. Data distribution was assessed for normality using the Shapiro–Wilk test. Comparisons were made between conservative and surgically managed mucocoeles for differences in demographic characteristics and outcomes in terms of intra-cranial and intra-orbital extension of the mucocoeles. Categorical variables were assessed using the χ^2^ test and continuous, non-normally distributed variables were assessed using the two-tailed Mann–Whitney U test. A *p*-value less than 0.05 was considered statistically significant.

### 2.7. Bias

Data from radiology reports were cross-checked with electronic clinic letters, discharge summaries, pathology results and operation notes to identify any inconsistencies. Discrepancies were discussed with the senior author (CP) where there was any uncertainty.

This project was a local service evaluation and did not require ethical approval, following completion of the NHS Research Ethics Committee Tool, provided by the Health Research Authority [15]. STROBE guidelines were adhered to for reporting [16].

## 3. Results

### 3.1. Participants

**Figure 1:**
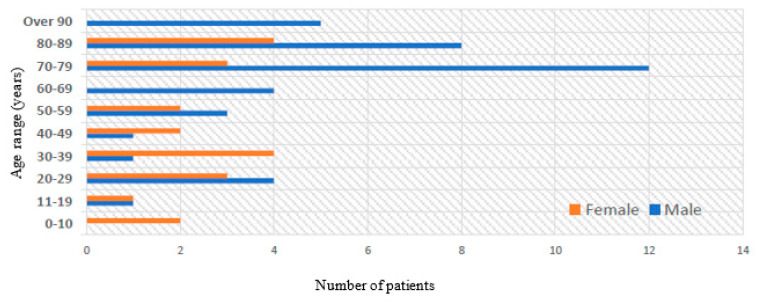
Age and gender of included patients.

Seventy-three unique patient records were screened for inclusion, of which 13 patients were excluded and sixty patients with mucocoeles were included; mean age 58 years (range 9–96); 65% male (Figure 1). Six patients (10%) had bilateral mucocoeles. Frontal and maxillary sinuses were most frequently affected. Seven patients were excluded for resolved mucocoeles, three for radiologically suspected mucocoeles, which were subsequently excluded clinically, two for nasolacrimal sac mucocoeles, and one for CRS without mucocoele.

A total of 45 cases were managed conservatively and 15 were managed surgically. The demographic characteristics of both groups are outlined in Table 1. Conservative and surgical mucocoeles did not differ in terms of age and sex of the patients or the sinuses involved.

### 3.2. Outcome Data

Thirty-five patients had incidental mucocoeles following a CT head scan from the emergency department or medical team for suspicion of head injury or cerebrovascular accident and were subsequently managed conservatively after the patient and/or images were reviewed by an Otorhinolaryngologist. The remainder of the patients had imaging requested by the Otorhinolaryngology, Ophthalmology or Maxillofacial surgery teams for outpatient investigation of symptoms that they presented with specifically due to the mucocoele. Data were incomplete for three patients (5%).

Overall, six patients (10%) had an intra-orbital extension of their mucocoele and three (5%) had an intra-cranial extension. There was no difference in rates of intra-cranial extension between conservative and surgical cases, but surgical cases included a higher rate of intra-orbital extension (27% vs. 4%, *p* = 0.01), (Table 2). One of 15 surgically managed patients presented with recurrence following surgery. This was a male patient in the 4th decade of life with a right frontal mucocoele, who initially underwent endoscopic drainage. The mucocoele re-accumulated in the right frontal sinus with communication to the right orbit and the patient subsequently underwent repeat endoscopic sinus surgery.

Examples of complicated mucocoeles included:

- Frontal mucocoele with extension into the antero-superior orbit causing orbital pain and headache, managed with a combined endoscopic and external approach.
- Frontal mucocoele invading the left orbit and exposing dura, managed with endoscopic sinus surgery and frontonasal anastomosis jointly with Ophthalmology.

One paediatric case was identified. This involved a 9-year-old with a resolved facial nerve palsy, intermittent nasal blockage and rhinorrhoea, who was identified as having an anterior ethmoid mucocoele on imaging, arising from a pneumatized uncinate process. This was managed surgically.

**Figure 2:**
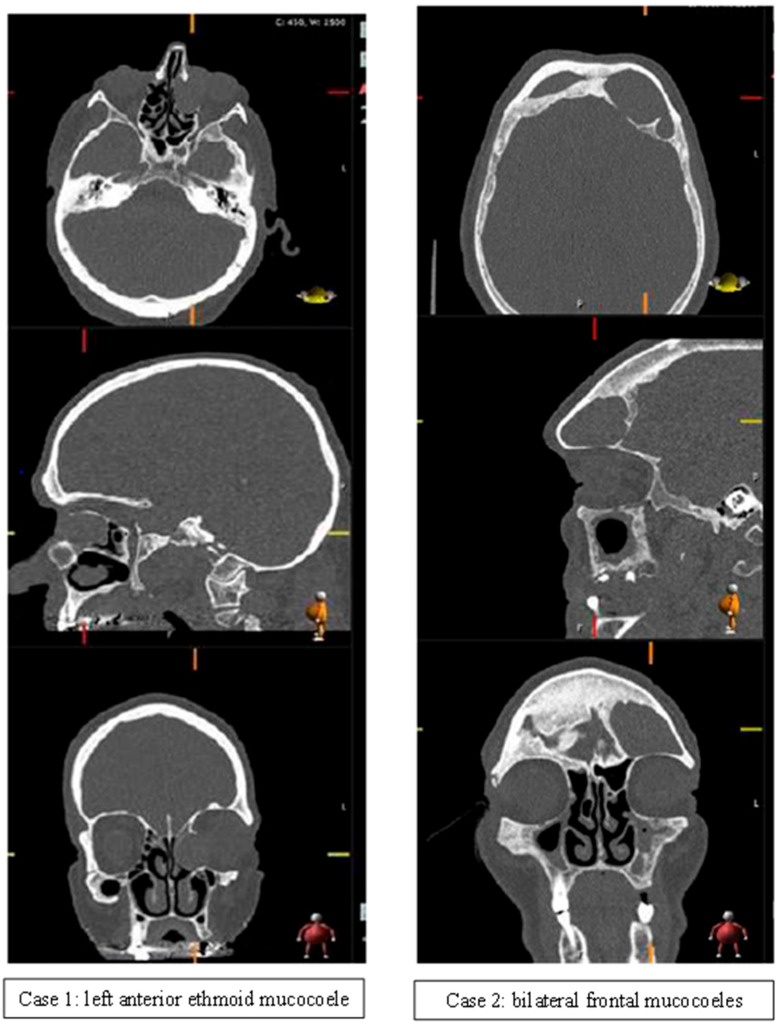
Case examples of conservatively managed mucocoeles.

Two examples of patients who declined the option of surgical management are outlined in Figure 2. Both patients were managed conservatively with repeat imaging due to patient choice. Case 1 demonstrates a left anterior ethmoid mucocoele with mass effect on the left orbit associated with post-nasal drip and medial canthus discharge. This was identified at 80 years of age and demonstrated unchanged appearances >10 years later following re-imaging for suspected head injury. The patient had no deterioration in symptoms during this time. Case 2 demonstrates bilateral frontal mucocoeles with extension into the left anterior cranial fossa, identified incidentally at 90 years of age following a head injury. Subsequent re-imaging 6 months later demonstrated no change. The patient was asymptomatic for 2.5 years following diagnosis.

## 4. Discussion

### 4.1. Key Results

The majority of mucocoeles in this study were asymptomatic and were incidental findings from radiological investigations for other clinical indications. These predominantly involved the frontal or maxillary sinuses, with three-quarters of patients not undergoing surgical management, and only 15% having an intra-orbital or intra-cranial extension. Most complicated cases were managed surgically and only one re-presented with recurrence. Surgical cases were more likely to be identified by the Otorhinolaryngology team as they were inherently symptomatic, and also had higher rates of intra-orbital, but not intra-cranial, extension compared to conservative cases. Where cases were managed conservatively due to patient choice, repeat imaging after several months showed no change in pathology. Whilst asymptomatic mucocoeles predominantly involved the maxillary sinus, symptomatic mucocoeles were most commonly identified in the frontal and sphenoid sinuses.

### 4.2. Interpretation and Comparison to Other Studies

These complication rates of extension beyond the sinuses, intra-cranially or intra-orbitally, are lower than other studies, including a seven-year review of 59 mucocoeles by Waizel-Haiat et al., which reported rates of 46% intra-orbital extension, 8% intra-cranial extension and 9% recurrence following surgery [9]. Similarly, Plantier et al. followed up 46 patients from 2005 to 2016 and identified rates of 43% orbital extension, 26% skull base extension and 15% recurrence following surgery [5]. However, both studies reported exclusively on surgically managed cases. Therefore, the rates of orbital and intra-cranial complications would have been higher as conservatively managed cases were excluded.

The mean age of patients at the time of diagnosis was 58 years old. This is comparable to previous reviews which have reported the greatest incidence of paranasal sinus mucocoeles between the fourth and sixth decades of life [17]. Several other primary studies have reported similar ages of presentation. A retrospective study by Lee et al. of 82 patients with extensive paranasal sinus mucocoeles, defined as mucocoeles with an intra-cranial and/or intra-orbital extension, reported a mean age at diagnosis of 52.7 years [18]. Similarly, a consecutive case series of 59 patients by Conboy et al. reported a mean age at diagnosis of 56 years; a retrospective study by Serrano et al. exploring surgical outcomes in 60 patients treated in France reported a mean age of 53 years; and a study by Scangas et al. investigating the natural history of 102 US patients found a mean age of 53 years at diagnosis [1,19,20]. Our study had an increased proportion of male patients compared to previous reviews. Overall, 65% of included patients were males and 35% females, although previous studies have reported an almost equal incidence of paranasal sinus mucocoeles between male and female patients [5,18,20]. This may have been due to the increased prevalence of trauma patients included in our study, since trauma patients are more likely to be male [21]. Moreover, this study found bilateral mucocoeles in 10% of patients, akin to the 12% reported by Conboy et al. [19].

This study found that the most frequently involved sinuses were the frontal and maxillary sinuses (each *n* = 21, 35%). A similar distribution was reported by Plantier et al., with 44% of mucocoeles involving the frontal sinus, followed by 20% involving the maxillary sinus [5]. Other studies, however, demonstrated the greatest frequency of involvement to be in the frontal and ethmoid sinuses, with the involvement of maxillary sinuses being uncommon. Conboy et al. reported 62% involvement of the frontal, 23% of the ethmoid and 9% of the maxillary sinuses [19]. Additionally, the proportion of sinus involvement in the study by Scangas et al. was 43% for frontal, 26% for ethmoid and 8% for maxillary sinuses [1]. Lee et al. displayed a low incidence of maxillary sinus mucocoeles of 9%, compared to 45% for frontal and 28% for ethmoid sinuses [18]. An important feature of these studies is that patients had profound morbidity at presentation, especially those with an intra-orbital and/or intra-cranial extension of the paranasal sinus mucocoeles. It is essential to highlight that the anatomical location of the maxillary sinus means that intra-orbital and intra-cranial extensions are rare; thus, there is a lower likelihood of patients presenting with these complications and having mucocoeles of maxillary origin. Maxillary sinus cases were more common in our study as they did not require referral to a tertiary neurosurgical unit and were more amenable to non-surgical management if indicated.

Our findings showed that surgically managed cases had higher rates of intra-orbital extension compared to conservative cases. This is to be expected as complicated mucocoeles with orbital symptoms require surgical management unless the patient declines this option. Although no difference was observed in rates of intra-cranial complications between both groups, this may have been due to the limited number of cases included in our study. Patients with an intra-cranial extension requiring emergency neurosurgical input may have presented directly to the local neurosurgical unit and therefore bypassed our local Otorhinolaryngology teams.

### 4.3. Strengths and Limitations

Our study included a follow-up period of up to 10 years and focussed on conservatively managed cases for which there is a paucity in the literature. However, our patient population may not be representative of a typical tertiary rhinology centre. Both hospitals included within our study do not have neurosurgical services on-site. Therefore, some cases of more severe mucocoeles may have been directly referred to a neurosurgical centre. We mitigated this by collating data on patients whose care was transferred out, but data on some patients (5%) were not retrievable. Additionally, patients may have been miscategorised if they were labelled with a mucocoele radiologically but were not referred to our Otorhinolaryngology service for thorough clinical follow-up and disease confirmation. Therefore, patients found to have alternative pathologies on further clinical investigation were excluded from this analysis.

### 4.4. Clinical Applicability and Generalisability

Local rhinology practice is for conservative management of asymptomatic mucocoeles, and patients with uncomplicated symptomatic mucocoeles are given the choice of surgical or conservative management. Complicated cases requiring surgery are managed with image guidance. If neurosurgical support is required, cases are discussed through the skull base multidisciplinary team fortnightly meeting. Since mucocoeles are relatively rare and lack established clinical practice guidelines, patients who underwent operative management received a diverse range of interventions with varying involvement of allied specialities such as Ophthalmology.

It is evident from this study that accurate guidelines are needed for the management of mucocoeles. Not all cases require surgical intervention, and further work is required to determine which cases should be operated on aside from those with complications or burdensome symptoms. This study suggests that the majority of asymptomatic mucocoeles may not cause any local complications within the short to medium term, but future prospective studies should investigate whether clinical or radiological parameters can predict which patients will develop complications. The most common symptom is headache, and this may be indistinguishable from facial pain and pressure in uncomplicated CRS [1]. This reinforces the need for imaging in recalcitrant CRS if endoscopic examination is insufficient.

The incidental, asymptomatic mucocoeles identified in this study demonstrated low complication rates. Additionally, the illustrated cases in this study demonstrate that even complicated mucocoeles that were identified incidentally had no change seen on serial imaging. Therefore, for incidental asymptomatic mucocoeles, an interval scan at 12 months may be suitable to identify those cases which are likely to become complicated. For those mucocoeles that demonstrate an increase in size at 12 months, additional serial imaging may be warranted.

The disadvantages associated with serial imaging for mucocoeles when compared to surgical management include the potential for patients becoming lost to follow-up and developing complications. Our study demonstrated multiple cases of asymptomatic mucocoeles which were identified incidentally, and these cases are likely to be most suitable for serial imaging. However, for patients who are unlikely to attend follow-up, the option of serial imaging must be considered with caution.

Although our study did not collect health economic data, serial imaging is associated with the healthcare costs of radiological investigations and clinical reviews. These must be compared to the costs of surgery, and future studies should include a health economic evaluation of the two management approaches to determine which is most cost-effective. Serial imaging is likely to be the more cost-effective approach if the proportion of patients who go on to require surgical management is low, as demonstrated in this study.

Due to the retrospective nature of this study and the limited number of centres, predictions about the behaviour of mucocoeles are somewhat limited. Future studies should involve long-term, prospective follow-up of mucocoeles from a diverse range of otorhinolaryngology centres. Outcomes such as need for surgery, symptom burden through patient-reported outcome measures and craniofacial cosmetic concerns should be considered alongside intra-cranial and intra-orbital complications. This would clarify the ideal time frame for serial imaging and the patient cohorts requiring closer follow-up. The clinical value of best practice measures such as serial clinical photographs and serial ophthalmological assessments should also be further investigated. Conservatively managed cases in this study had varying adherence to follow-up and so the optimum clinical pathway for these patients remains uncertain.

Future studies should also explore the effect of risk factors for mucocoeles, such as prior surgery, on clinical outcomes. In the retrospective review of 102 patients by Scangas et al., authors found that patients with prior endoscopic sinus surgery developed paranasal sinus mucocoeles sooner than those with other risk factors such as prior maxillofacial trauma or open surgery [1]. The mean time for mucocoele development in those who had endoscopic sinus surgery in the past was 5.3 years, compared to 17.7 years and 18.1 years for those with a prior history of maxillofacial trauma with no surgery and those with prior open surgery, respectively. This finding was analogous to the retrospective study of 60 patients in France by Serrano et al., which reported that mucocoele development had an interval of 18 years for those with prior open surgery in contrast to 2 years for those with a history of prior endoscopic sinus surgery [20]. The authors hypothesised that this relationship was due to the association of endoscopic sinus surgery with the presence of chronic rhinosinusitis, which is an independent risk factor for the formation of paranasal sinus mucocoele. Future studies should therefore investigate the independent relationship between causative factors such as prior surgery with the clinical course of paranasal sinus mucocoeles.

## 5. Conclusions

We highlight the varying clinical course of mucocoeles and identify local complication rates for predominantly incidental paranasal sinus mucocoeles. Conservative management with serial imaging was sufficient for the majority of uncomplicated mucocoeles in this study, but further prospective studies are required to determine which cases would benefit from early operative intervention.

## Figures and Tables

**Table 1:** Characteristics of included patients.

	Conservatively Managed Mucocoeles	Surgically Managed Mucocoeles	*p*-Value
*n*	45	15	-
Mean age (SD)	64.4 (24.8)	51.9 (25.1)	0.08
Male (%)	31 (69)	8 (53)	0.27
Sinus involved (%): Maxillary	19 (42)	2 (13)	0.23
Ethmoid	5 (11)	3 (20)	
Frontal	14 (31)	7 (47)	
Sphenoid	9 (20)	4 (27)	

Key: SD = standard deviation.

**Table 2:** Management and outcomes of mucocoeles.

	Conservatively Managed Mucocoeles	Surgically Managed Mucocoeles	*p*-Value
Source of data (%)			<0.01
Identified by Otolaryngology team	8 (18)	15 (100)	
Identified incidentally	35 (78)	0 (0)	
Lost to follow-up	2 (4)	1 (7)	
Intra-cranial extension (%)	1 (2)	2 (13)	0.09
Intra-orbital extension (%)	2 (4)	4 (27)	0.01
Recurrence (%)	- (-)	1 (7)	-

## Data Availability

The data presented in this study are available on request from the corresponding author.

## References

[1] Scangas G.A., Gudis D.A., Kennedy D.W. (2013). The natural history and clinical characteristics of paranasal sinus mucoceles: A clinical review. Int. Forum Allergy Rhinol..

[2] Rastin M.S., Rasouly N. (2023). Giant ethmoid mucocele presenting as a nasal region mass with intracranial extension, a case report. Radiol. Case Rep..

[3] Thompson L.D.R. (2012). Paranasal sinus mucocele. Ear Nose Throat J..

[4] Devars Du Mayne M., Moya-Plana A., Malinvaud D., Laccourreye O., Bonfils P. (2012). Sinus mucocele: Natural history and long-term recurrence rate. Eur. Ann. Otorhinolaryngol. Head Neck Dis..

[5] Plantier D.B., Neto D.B., De Rezende Pinna F., Voegels R.L. (2019). Mucocele: Clinical characteristics and outcomes in 46 operated patients. Int. Arch. Otorhinolaryngol..

[6] Sadiq S.A., Lim M.K., Jones N.S. (2009). Ophthalmic manifestations of paranasal sinus mucocoeles. Int. Ophthalmol..

[7] Swain S.K., Dubey D. (2023). Paranasal Sinus Mucoceles: A Narrative Review. Hamdan Med. J..

[8] Dos Santos P.L., Chihara L.L., Alcalde L.F.A., Masalskas B.F., Sant’ana E., Faria P.E.P. (2017). Outcomes in Surgical Treatment of Mucocele in Frontal Sinus. J. Craniofac. Surg..

[9] Waizel-Haiat S., Díaz-Lara I.M., Vargas-Aguayo A.M., Santiago-Cordova J.L. (2017). Experience in the surgical treatment of paranasal sinus mucoceles in a university hospital. Cir. Cir. Engl. Ed..

[10] Iseh K. (2010). Endoscopic and external surgical approach to paranasal sinus mucocele. J. Surg. Tech. Case Rep..

[11] Fokkens W.J., Lund V.J., Hopkins C., Hellings P.W., Kern R., Reitsma S., Toppila-Salmi S., Bernal-Sprekelsen M., Mullol J., Alobid I. (2020). European Position Paper on Rhinosinusitis and Nasal Polyps 2020. Rhinology.

[12] Woodworth B.A., Harvey R.J., Neal J.G., Palmer J.N., Schlosser R.J. (2008). Endoscopic management of frontal sinus mucoeceles with anterior table erosion. Rhinology.

[13] Soon S.R., Lim C.M., Singh H., Sethi D.S. (2010). Sphenoid sinus mucocele: 10 cases and literature review. J. Laryngol. Otol..

[14] Miller C., Berens A., Patel S.A., Humphreys I.M., Moe K.S. (2019). Transorbital Approach for Improved Access in the Management of Paranasal Sinus Mucoceles. J. Neurol. Surg. Part B Skull Base.

[15] Do I Need NHS Ethics Approval?. https://www.hra-decisiontools.org.uk/ethics/.

[16] von Elm E., Altman D.G., Egger M., Pocock S.J., Gøtzsche P.C., Vandenbroucke J.P. (2007). The Strengthening the Reporting of Observational Studies in Epidemiology (STROBE) statement: Guidelines for reporting observational studies. Lancet.

[17] Bockmühl U., Kratzsch B., Benda K., Draf W. (2005). Paranasal sinus mucoceles: Surgical management and long term results. Laryngorhinootologie.

[18] Lee T.J., Li S.P., Fu C.H., Huang C.C., Chang P.H., Chen Y.W., Chen C.W. (2009). Extensive paranasal sinus mucoceles: A 15-year review of 82 cases. Am. J. Otolaryngol..

[19] Conboy P.J., Jones N.S. (2003). The place of endoscopic sinus surgery in the treatment of paranasal sinus mucocoeles. Clin. Otolaryngol. Allied Sci..

[20] Serrano E., Klossek J.M., Percodani J., Yardeni E., Dufour X. (2004). Surgical management of paranasal sinus mucoceles: A long-term study of 60 cases. Otolaryngol. Head Neck Surg..

[21] Moran C.G., Lecky F., Bouamra O., Lawrence T., Edwards A., Woodford M., Willett K., Coats T.J. (2018). Changing the System—Major Trauma Patients and Their Outcomes in the NHS (England) 2008–2017. eClinicalMedicine.

